# Acute Biochemical Responses to Competitive Tournament Load in Female Handball Players: Hormonal, Inflammatory and Muscle Damage Markers

**DOI:** 10.3390/life16030523

**Published:** 2026-03-21

**Authors:** Zarife Pancar, Yücel Makaracı, Celal Gençoğlu, Burak Karaca, Hasan Ulusal

**Affiliations:** 1 Department of Physical Education and Sports, Faculty of Sports Science, Gaziantep University, Gaziantep 27350, Türkiye; 2 Department of Coaching Education, Faculty of Sports Sciences, Karamanoğlu Mehmetbey University, Karaman 70100, Türkiye; yucelmkrc@gmail.com; 3 Department of Physical Education and Sports Teaching, Faculty of Sport Sciences, Dokuz Eylül University, İzmir 35390, Türkiye; celal.gencoglu@deu.edu.tr; 4 Institute of Health Sciences, Department of Physical Education and Sports, Gaziantep University, Gaziantep 27310, Türkiye; burakkaracapt@gmail.com; 5 Department of Medical Biochemistry, Faculty of Medicine, Gaziantep Islam Science and Technology University, Gaziantep 27010, Türkiye; hasan_ulusal@hotmail.com

**Keywords:** handball, female athletes, hormonal response, inflammation, muscle damage

## Abstract

Background: Congested tournament schedules impose substantial physiological stress in team sports; however, the integrated endocrine and inflammatory responses to real competitive match load in female handball players remain insufficiently characterized. Objective: This study aimed to characterize the acute biochemical responses, including hormonal, inflammatory, muscle damage, and bone metabolism markers, elicited by competitive tournament load in female handball players and to provide practical insights for optimizing recovery strategies and load management during short-term competitive periods. Methods: In a pre–post study design, venous blood samples were collected from competitive female athletes (n = 8; age 20.83 ± 2.93 years) before the first match and after the fourth consecutive match of an official university qualification tournament. Biochemical analyses included cortisol, insulin, IL-6, creatine kinase (CK), IGF-1, irisin, lactate dehydrogenase (LDH), osteocalcin, and testosterone. Pre-to-post changes were assessed using paired *t*-tests and effect sizes. Results: Tournament load induced substantial multisystem physiological perturbations. Significant increases were observed in cortisol (*p* < 0.001), insulin (*p* = 0.044), IL-6 (*p* < 0.001), CK (*p* < 0.001), and osteocalcin (*p* = 0.005), indicating activation of the hypothalamic–pituitary–adrenal axis, systemic inflammation, muscle membrane disruption, and enhanced bone turnover. Conversely, IGF-1 (*p* < 0.001) and testosterone (*p* = 0.004) significantly decreased, reflecting suppression of anabolic signaling and a shift toward a catabolic hormonal environment under cumulative match stress. LDH significantly decreased (*p* = 0.002), while irisin showed no significant change (*p* > 0.05). Conclusions: These findings demonstrate that congested tournament schedules provoke an integrated endocrine–inflammatory stress response in female handball players. Importantly, the observed anabolic–catabolic imbalance highlights the need for individualized recovery strategies, optimized load management, and adequate recovery periods to mitigate maladaptation and reduce injury risk during short-term competitive tournaments.

## 1. Introduction

Handball is a high-intensity, multidimensional team sport characterized by repeated sprinting, rapid accelerations and decelerations, explosive changes in direction, forceful throwing actions, and frequent physical contact. This demanding activity profile imposes substantial metabolic, neuromuscular, and endocrine stress on athletes, requiring continuous integration of anaerobic and aerobic energy systems [1,2]. Tournament formats, in which multiple matches are played within a condensed time frame, further amplify this physiological burden. The accumulation of high-intensity efforts combined with limited inter-match recovery intervals can disrupt endocrine homeostasis, intensify systemic inflammatory responses, and exacerbate exercise-induced muscle damage. Collectively, these factors increase the risk of acute fatigue, impair neuromuscular performance, and delay recovery [3,4]. Such congested competitive schedules may therefore provoke a coordinated multisystem stress response that extends beyond isolated metabolic strain.

During high-tempo tournament periods, the marked increase in physiological load activates the hypothalamic–pituitary–adrenal (HPA) axis, resulting in elevated cortisol secretion. Cortisol plays a central role in the regulation of metabolic stress by stimulating gluconeogenesis, lipolysis, and protein catabolism, thereby ensuring adequate energy availability under repeated high-intensity demands [5,6,7]. While this response is essential for short-term performance maintenance, sustained HPA axis activation during congested competition may shift the anabolic–catabolic balance toward a more catabolic hormonal environment. Concurrently, increases in myokines such as interleukin-6 (IL-6) reflect not only an amplified inflammatory response but also the cytokine’s dual role in coordinating immune signaling and energy metabolism during exercise [8,9,10,11,12]. IL-6 is rapidly released from contracting skeletal muscle and contributes to glucose homeostasis and substrate mobilization, linking local muscular stress to systemic metabolic regulation. In high-impact sports such as handball, characterized by substantial eccentric loading, repeated sprinting, and frequent physical contact, elevations in muscle damage biomarkers—including creatine kinase (CK) and lactate dehydrogenase (LDH)—indicate compromised sarcolemma integrity and mechanical strain at the muscular level [13,14]. Together, these endocrine, inflammatory, and structural responses illustrate the coordinated multisystem adaptation elicited by repeated competitive match exposure.

Conversely, anabolic hormonal responses may be attenuated during congested tournament periods, with reductions in insulin-like growth factor-1 (IGF-1) and testosterone impairing muscle protein synthesis, tissue remodeling, and recovery capacity [15,16]. A sustained decrease in these anabolic mediators, particularly when accompanied by elevated cortisol, reflects a disruption of the anabolic–catabolic equilibrium that is essential for optimal neuromuscular adaptation and performance maintenance. Such endocrine shifts may compromise muscle repair, reduce force-generating capacity, and delay functional recovery following repeated high-intensity match exposure. This suppression may be especially pronounced in female athletes, whose hormonal milieu is influenced by menstrual cycle phase, energy availability, and heightened stress sensitivity. Compared with males, women exhibit distinct regulatory interactions between the hypothalamic–pituitary–gonadal and hypothalamic–pituitary–adrenal axes, which may render them more vulnerable to endocrine perturbations during periods of accumulated competitive stress [17,18]. Furthermore, low energy availability and short recovery intervals may exacerbate anabolic suppression, potentially increasing the risk of maladaptation and injury. Understanding how short-term tournament loads modulate the integrated endocrine–inflammatory profile in female athletes is therefore critical for preserving performance capacity, optimizing recovery strategies, and safeguarding long-term health.

Despite growing interest in workload monitoring in team sports, the acute biochemical consequences of congested official tournament match play in female handball players remain insufficiently explored. Most existing research has focused on isolated matches, laboratory simulations, or male cohorts, leaving a limited understanding of the integrated endocrine–inflammatory responses elicited under authentic competitive conditions in female athletes. Accordingly, a comprehensive evaluation of the hormonal, inflammatory, muscle damage, and bone metabolism responses to consecutive official matches addresses a meaningful gap in both scientific literature and applied sport practice. Characterizing these multisystem physiological alterations may provide valuable insights for coaches, performance scientists, and sports medicine practitioners seeking to optimize load management, recovery protocols, and injury prevention strategies during short-term tournament schedules. Therefore, the aim of this study was to characterize the acute biochemical responses induced by four successive matches during an interuniversity women’s handball tournament, thereby delineating the integrated physiological stress profile associated with real-world competitive demands in female athletes.

## 2. Materials and Methods

### 2.1. Experimental Approach to the Study

This study employed a single-group pre–post experimental design to examine the acute biochemical responses induced by a congested official tournament match schedule in female handball players. This design was selected to allow each athlete to serve as her own control, thereby reducing inter-individual variability while preserving the ecological validity of authentic competitive conditions—an important consideration in elite team sport research where experimental manipulation of match load is not feasible. Measurements were conducted at two standardized time points: (i) prior to the first match of the tournament, following at least 24 h of rest to establish a baseline resting state; and (ii) 24 h after the fourth consecutive match, reflecting the cumulative physiological impact of repeated match exposure. This timing was selected to capture the cumulative and integrated physiological response to tournament load rather than immediate transient post-match fluctuations.

**Figure 1:**
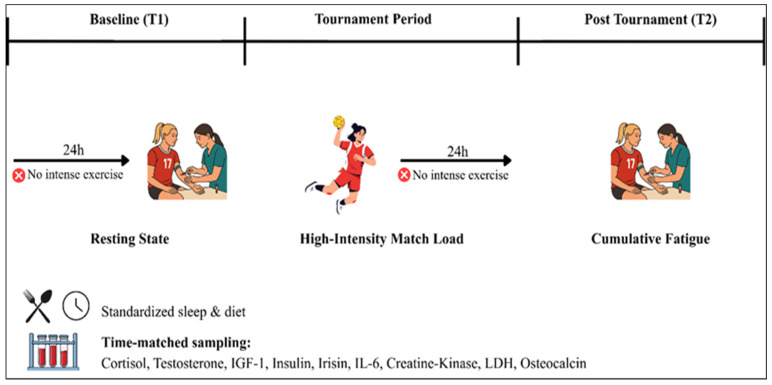
Study Flow Diagram.

All assessments were performed within the same morning time window to control for circadian variation in hormonal secretion. Participants were instructed to refrain from strenuous physical activity for 24 h prior to baseline sampling. Participants were instructed to maintain their habitual nutrition, hydration, and sleep patterns throughout the tournament period. The comprehensive biochemical panel included key endocrine (cortisol, testosterone, IGF-1, insulin, irisin), inflammatory (IL-6), muscle damage (CK), and bone metabolism (osteocalcin) markers. The absence of a control group reflects the practical constraints inherent to real competition settings, where tournament scheduling cannot be manipulated. To mitigate this limitation and strengthen internal validity, paired comparisons and effect size calculations were performed. The experimental design of the study is illustrated in Figure 1.

Given the ecological nature of the study conducted during an official tournament, the inclusion of a traditional control group was not feasible. Instead, a within-subject repeated-measures design was adopted, allowing each participant to serve as her own control. This approach is widely accepted in sport science research under real competitive conditions, where external standardization of match load is not possible. Additionally, a post hoc power analysis was conducted using G*Power (Version 3.1.9.7, Heinrich Heine University, Düsseldorf, Germany). Based on the observed large effect size for cortisol (d = 3.699), with a sample size of n = 8 and α = 0.05, the statistical power (1 − β) was calculated as 0.99, indicating a very high level of sensitivity.

Ethical approval was obtained from the Gaziantep University Health and Sports Ethics Committee (Decision No: 2025-01; Date: 15 April 2025), and all procedures were conducted in accordance with the Declaration of Helsinki. Written informed consent was obtained from all participants prior to study participation.

### 2.2. Participants

Participants were recruited during the Interuniversity Women’s Handball Qualification Tournament held in May. All active players included in the official tournament roster were enrolled in the study to preserve ecological validity. A total of eight competitive female handball players participating in the Interuniversity Women’s Handball Qualification Tournament voluntarily took part in this study (descriptive characteristics are presented in Table 1). The sample comprised the entire active tournament roster of the team, and thus a purposive sampling strategy was applied to preserve ecological validity within a real competitive setting. All participants had a minimum of five years of competitive handball experience, trained at least four times per week, and were free from any acute or chronic medical conditions. Inclusion criteria required athletes to be free from musculoskeletal injury, surgical intervention, or medical treatment within the preceding three months, to abstain from ergogenic aids and anti-inflammatory medications, and to have maintained uninterrupted participation in regular training. Given the known influence of hormonal fluctuations on endocrine and inflammatory biomarkers, menstrual cycle considerations were incorporated into participant screening. Athletes with acute illness, diagnosed hormonal or metabolic disorders, or those assessed during transitional menstrual phases were excluded from biochemical analysis to minimize physiological variability. All participants received detailed verbal and written information regarding study procedures and provided written informed consent prior to enrollment.

### 2.3. Tournament Structure and Competitive Load

Participants competed in an interuniversity qualification tournament consisting of four matches played over four consecutive days. Each match followed official international handball regulations (2 × 30 min with a 10-min halftime interval). Venous blood samples were collected under standardized resting conditions in the morning, 24 h prior to the first match, representing baseline values. The second blood sample was obtained 24 h after the fourth consecutive match to capture the cumulative physiological impact of repeated competitive exposure. This timing was selected to reflect integrated biochemical responses to tournament load rather than acute transient fluctuations immediately following match play. Participants competed according to tactical strategies and rotation plans determined by the coaching staff. Although total playing time and positional responsibilities varied according to match-specific demands, all athletes were regularly involved in match play throughout the tournament period. No additional training sessions, resistance exercises, or high-intensity conditioning activities were performed during the tournament, thereby preventing external training load from influencing physiological outcomes. Internal load was monitored using the Borg CR-10 Rating of Perceived Exertion (RPE) scale [19]. Athletes reported their perceived exertion immediately following each match. RPE values demonstrated a progressive increase across the tournament (Day 1: 4–5; Day 2: 5–6; Day 3: 7–8; Day 4: ≥9), indicating progressive accumulation of internal load and perceived fatigue across consecutive competition days.

To minimize confounding influences, participants were instructed to maintain their habitual dietary intake, hydration practices, and sleep routines throughout the tournament. The use of ergogenic aids, nutritional supplements, or pharmacological agents was strictly prohibited. This controlled competitive environment strengthens the interpretation that observed biochemical alterations were primarily driven by internal load accumulation and repeated high-intensity match demands. As all matches were conducted at full duration under official international regulations within a high-stakes qualification context, the physiological stressors reflected authentic competitive conditions. This methodological approach enhances ecological validity and provides a realistic representation of biochemical responses to congested tournament play in female handball athletes.

### 2.4. Blood Sampling and Biochemical Analyses

For each measurement point, approximately 5 mL of venous blood was collected from the antecubital vein under fasting conditions between 08:00 and 09:00 to control for circadian variation in hormonal secretion. Blood samples were collected into serum separator tubes and immediately centrifuged at 4000 rpm for 10 min at 4 °C. The separated serum was aliquoted into sterile microtubes and stored at −80 °C until biochemical analysis. All samples from both time points were analyzed within the same analytical batch to minimize inter-assay variability. Serum concentrations of cortisol, insulin, IGF-1, testosterone, IL-6, osteocalcin, irisin, CK, and LDH were quantified using commercially available enzyme-linked immunosorbent assay (ELISA) kits (FineTest^®^, Wuhan Fine Biotech Co., Ltd., Wuhan, China) according to the manufacturer’s instructions [20]. All samples were analyzed in duplicate, and the mean value of the two measurements was used for statistical analysis. Analytical reliability was confirmed through intra-assay and inter-assay coefficients of variation (CV), which were maintained below 10% for all biomarkers, indicating acceptable assay precision.

### 2.5. Statistical Analyses

All statistical analyses were performed using Jamovi (Version 2.5.6, Sydney, Australia) and GraphPad Prism (Version 10.3.1, GraphPad Software, San Diego, CA, USA). Descriptive statistics for each variable are reported as mean ± standard deviation (SD), along with 95% confidence intervals (CI) for the mean. Normality of the data was assessed visually through Q-Q plots and confirmed analytically via the Shapiro–Wilk test. To examine the pre- and post-intervention differences in hormonal, inflammatory, and biochemical markers, paired samples *t*-tests were conducted for each dependent variable. Effect sizes were calculated using Cohen’s d, and interpreted according to conventional thresholds (small: 0.2; medium: 0.5; large: 0.8) [21,22]. The precision of the effect size estimates is presented using 95% confidence intervals. A two-tailed *p*-value of less than 0.05 was considered statistically significant. Data visualization (e.g., bar plots with error bars, individual change plots) and supplementary comparisons were conducted in GraphPad Prism to illustrate individual and group-level changes across time points.

## 3. Results

The descriptive characteristics of the participants are presented in Table 1. The distributions of age, height, body mass, and training experience indicate that the sample represents a homogeneous group of competitive athletes with comparable anthropometric profiles and similar sporting backgrounds.

Table 1 reports the baseline descriptive statistics for participant demographics and physical characteristics. The participants had a mean age of 21.75 years (SD = 1.39, 95% CI [20.589, 22.911]) and a training age of 7.5 years (SD = 1.31, 95% CI [6.405, 8.595]), indicating a relatively well-trained sample. The average height was 1.657 m (SD = 0.056, 95% CI [1.611, 1.704]), and the average weight was 57.25 kg (SD = 6.02, 95% CI [52.219, 62.281]). BMI values suggest that participants were within a normal weight range, with a mean BMI of 20.83 kg/m^2^ (SD = 1.82, 95% CI [19.309, 22.358]).

Table 2 presents the descriptive and inferential statistics for hormonal, inflammatory, and biochemical markers measured before and after the intervention. Significant increases were observed in cortisol, insulin, IL-6, CK, and osteocalcin (*p* < 0.05 for all). In contrast, IGF-1 and testosterone levels significantly decreased following the intervention (*p* < 0.05). LDH also showed a significant decrease (*p* < 0.05), while irisin levels did not change significantly (*p* > 0.05).

**Figure 2:**
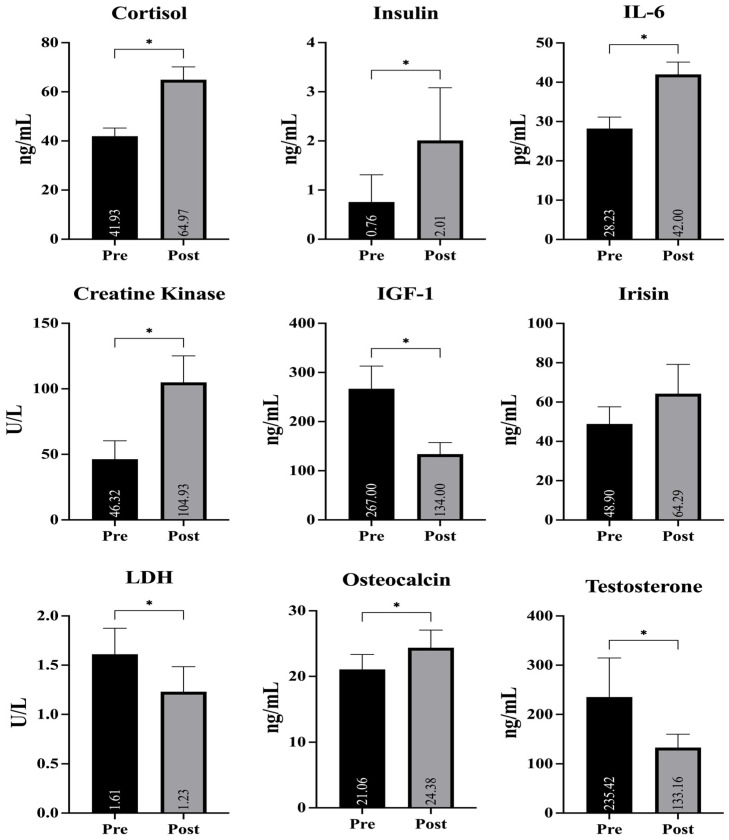
Pre- and post-tournament changes in hormonal, inflammatory, muscle damage, and bone metabolism biomarkers in female handball players * indicates *p* < 0.05.

As illustrated in Figure 2, the tournament induced substantial alterations across multiple biochemical markers, including significant increases in cortisol, IL-6, CK, and osteocalcin, alongside reductions in IGF-1 and testosterone.

## 4. Discussion

This study provides one of the few comprehensive evaluations of acute biochemical responses to a congested four-match competitive schedule completed over four consecutive days in female handball players. The principal findings indicate that short recovery intervals inherent to tournament formats provoke a coordinated multisystem physiological stress response, characterized by marked alterations in endocrine regulation, systemic inflammation, muscle membrane integrity, and bone metabolism. Specifically, the observed elevation in cortisol and IL-6, accompanied by increases in CK and osteocalcin and reductions in IGF-1 and testosterone, reflects a shift toward a catabolic hormonal milieu under cumulative competitive stress. Handball’s high-intensity intermittent profile—encompassing repeated sprinting, abrupt accelerations and decelerations, explosive directional changes, and frequent physical contact—imposes substantial metabolic and mechanical strain [23,24,25]. Within a congested tournament structure, insufficient between-match recovery may amplify fatigue accumulation, disrupt endocrine–cytokine homeostasis, and exacerbate exercise-induced muscle damage. By capturing responses under authentic competitive conditions, the present study offers strong ecological validity and delineates an integrated stress-response profile that laboratory-based simulations may fail to fully reproduce. Importantly, given the known sex-specific differences in hormonal regulation, energy availability, and stress reactivity [26,27], these findings provide valuable insight into the physiological vulnerability of female athletes during short-term congested tournament exposure.

The significant increase in cortisol observed in this study indicates activation of the HPA axis in response to cumulative tournament load. Cortisol plays a key role in metabolic regulation by supporting energy availability during repeated high-intensity efforts. However, sustained elevation 24 h after the final match suggests incomplete physiological recovery and a persistent catabolic response. Back-to-back competitions in team sports have been shown to induce increases in cortisol levels, reflecting both acute energy mobilization and adaptation to competitive stress [28]. In handball, the combination of repeated physical contact, eccentric loading, and high metabolic demand may further contribute to this response during congested tournament schedules [29,30]. Prolonged cortisol elevation may influence the anabolic–catabolic balance by interacting with testosterone and IGF-1 responses, potentially affecting recovery processes. In this context, the concurrent increase in perceived exertion across the tournament supports the presence of cumulative fatigue. Borg CR-10 ratings increased progressively from moderate levels at the beginning of the tournament to very high values following the final match, indicating increased internal load and incomplete recovery. Together, these findings suggest that congested tournament formats impose substantial physiological demands, with potential implications for recovery management and performance maintenance.

The significant reductions in IGF-1 and testosterone observed in the present study indicate a suppression of anabolic signaling and a transient inhibition of the hypothalamic–pituitary–gonadal (HPG) axis under cumulative tournament stress. Both IGF-1 and testosterone are central regulators of muscle protein synthesis, satellite cell activation, myofibrillar repair, and tissue remodeling. A decline in these anabolic mediators—particularly when accompanied by sustained cortisol elevation—reflects a disruption of the testosterone/IGF-1–to–cortisol balance, favoring a catabolic endocrine environment [16,31,32]. Such endocrine alterations may be especially pronounced in female athletes. Sex-specific differences in hormonal regulation, energy availability, and stress reactivity may render women more vulnerable to anabolic suppression during periods of accumulated competitive load [33]. Low energy availability, short recovery intervals, and repeated high-intensity exposure can exacerbate this imbalance, potentially accelerating fatigue development and impairing adaptive remodeling. Consistent with our findings, previous research in female handball players has reported significant reductions in IGF-1 and testosterone during congested competition schedules [29]. From a functional perspective, the combined elevation of cortisol and reduction in anabolic hormones may slow muscle protein synthesis, delay structural repair following eccentric loading, and compromise neuromuscular recovery. Consequently, athletes may exhibit increased muscle soreness, reduced reactive strength, diminished jump performance, impaired repeated-sprint capacity, and slower change-of-direction ability. Over short recovery cycles, this hormonal milieu may contribute to cumulative fatigue and increase susceptibility to performance decrement and musculoskeletal injury.

Sustained activation of the HPA axis during periods of accumulated physiological stress may disturb autonomic nervous system balance, potentially leading to reductions in heart rate variability, a recognized marker of elevated fatigue and delayed parasympathetic recovery [34]. The hormonal pattern observed in the present study—characterized by elevated cortisol alongside reduced testosterone and IGF-1—appears consistent with the early biological profile associated with functional overreaching in athletes [35,36,37]. Although overtraining cannot be directly inferred from the present design, the observed anabolic–catabolic imbalance suggests a transient reduction in recovery capacity under congested competitive load. Such endocrine alterations may be associated with increased fatigue and impaired neuromuscular function during short recovery cycles. Given the high physical demands of handball, these responses may have practical implications for performance maintenance and recovery management.

The significant increase in osteocalcin observed in the present study likely reflects the stimulatory effect of repeated mechanical loading on bone turnover during congested tournament play. Handball involves frequent high-impact actions—including jumping, rapid directional changes, and physical contact—which impose substantial mechanical strain on the skeletal system. Such loading stimuli are known to accelerate bone remodeling dynamics and enhance osteoblastic activity, leading to elevated circulating osteocalcin concentrations [38,39]. In tournament settings characterized by repeated match exposure within a short timeframe, cumulative mechanical strain may therefore contribute to increased bone turnover responses. In addition to its role as a marker of bone formation, osteocalcin has been associated with metabolic regulation, particularly in relation to glucose metabolism and insulin sensitivity [40,41]. In this context, the concurrent increase in osteocalcin and insulin observed in the present study may reflect a coordinated physiological response to increased metabolic demand during congested competition. Given the complex interaction between energy availability, hormonal fluctuations, and bone metabolism in female athletes, such responses may have implications for both recovery processes and physiological adaptation. However, the potential impact of accelerated bone turnover during short recovery cycles warrants careful monitoring, particularly in relation to long-term skeletal health [39].

The significant increase in insulin observed in the present study may reflect a physiological response to the high energetic demands and cumulative glycogen depletion associated with a congested tournament schedule. In high-intensity intermittent sports such as handball, repeated sprinting and rapid changes in movement can lead to substantial reductions in muscle glycogen stores. When matches are performed on consecutive days, incomplete glycogen restoration may occur, potentially stimulating compensatory metabolic responses aimed at accelerating substrate replenishment. Post-exercise elevations in insulin are well recognized as key mediators of glucose uptake and glycogen resynthesis [42,43]. In this context, the elevated insulin levels observed after the final match may indicate an increased metabolic demand to support recovery processes under accumulated physiological stress. Notably, this response occurred alongside elevated cortisol and reduced IGF-1 and testosterone levels, suggesting a transient imbalance between anabolic and catabolic processes. In female athletes, the interaction between energy availability and hormonal responses is particularly complex [44,45]. Although energy availability was not directly assessed, this hormonal pattern may reflect a temporary disruption in recovery capacity during congested competition [32,46].

This study has several limitations that warrant consideration. First, the sample size was modest, reflecting the inclusion of the full competitive roster within a real tournament setting. While this may restrict statistical generalizability, the within-subject design and high ecological validity strengthen the internal consistency and practical relevance of the findings. Second, biochemical measurements were obtained at two time points, which limits insight into the precise temporal evolution of endocrine and inflammatory responses across each match and recovery interval. More frequent sampling could provide a clearer understanding of short-term recovery kinetics. Although internal load was monitored using the Borg CR-10 scale, objective external load metrics (e.g., GPS-derived data) and comprehensive performance measures were not incorporated. Therefore, direct associations between biochemical stress markers, mechanical workload, and functional performance outcomes could not be fully established. Finally, the present results reflect physiological responses to a specific four-match tournament structure. Variations in match density, seasonal phase, training status, and athlete conditioning may produce different adaptive or maladaptive patterns. Future investigations employing larger cohorts, multidimensional load monitoring, and longitudinal follow-up designs are warranted to further elucidate the interaction between competitive load, endocrine balance, and recovery capacity in female handball athletes. From a practical perspective, the findings of this study provide important implications for training and recovery management in female handball players during congested tournament periods. Coaches and practitioners should consider implementing individualized recovery strategies, including optimized sleep, nutritional support, and active recovery protocols, particularly following consecutive match days. Monitoring hormonal and biochemical markers may help identify early signs of cumulative fatigue and anabolic suppression, enabling timely adjustments in training load. Furthermore, strategic player rotation and load distribution during tournaments may be critical to minimize physiological strain and reduce the risk of performance decline and injury.

## 5. Conclusions

This study demonstrates that a congested four-match tournament schedule elicits a pronounced physiological stress response in female handball players. Elevated cortisol alongside reductions in IGF-1 and testosterone indicate a shift toward a catabolic hormonal state, suggesting a transient reduction in recovery capacity. Concurrent increases in IL-6 and CK reflect inflammatory activity and muscle stress, while elevations in insulin and osteocalcin may indicate adaptive metabolic responses to cumulative energetic demand. Collectively, these findings highlight the substantial physiological demands imposed by congested tournament schedules. From a practical perspective, these results emphasize the importance of individualized load management, strategic player rotation, and optimized recovery strategies during short-term competitive periods. Such approaches may support performance maintenance and recovery under conditions of repeated match exposure.

## Figures and Tables

**Table 1:** Descriptive Statistics and 95% Confidence Intervals for Participant Characteristics.

Variable	Mean	95% CI Lower	95% CI Upper	SD
Age (years)	21.750	20.589	22.911	1.389
Height (m)	1.657	1.611	1.704	0.056
Weight (kg)	57.250	52.219	62.281	6.018
Training Age (years)	7.500	6.405	8.595	1.309
BMI (kg/m^2^)	20.834	19.309	22.358	1.824

Notes. Values are presented as mean ± standard deviation (SD) with 95% confidence intervals (CIs). BMI: Body Mass Index.

**Table 2:** Descriptive Statistics and Paired Samples *t*-Test Results for Hormonal, Inflammatory, and Biochemical Markers (Pre- and Post-Test).

Variable	Pre (M ± SD)	Post (M ± SD)	t	*p*	d	95% CI
Cortisol (ng/mL)	41.931 ± 3.343	64.965 ± 5.196	−10.462	**<0.001**	−3.699	[−5.704, −1.676]
Insulin (ng/mL)	0.757 ± 0.554	2.009 ± 1.072	−2.459	**0.044**	−0.869	[−1.673, −0.024]
IL-6 (pg/mL)	28.229 ± 2.903	41.995 ± 3.134	−13.750	**<0.001**	−4.861	[−7.436, −2.279]
Creatine Kinase (U/L)	46.316 ± 14.076	104.931 ± 20.189	−6.477	**<0.001**	−2.290	[−3.634, −0.910]
IGF-1 (ng/mL)	267.000 ± 46.056	134.000 ± 23.336	7.056	**<0.001**	2.495	[1.026, 3.931]
Irisin (ng/mL)	48.901 ± 8.667	64.286 ± 14.870	−2.073	0.077	−0.733	[−1.502, 0.075]
LDH (U/L)	1.611 ± 0.264	1.230 ± 0.254	4.892	**0.002**	1.730	[0.583, 2.834]
Osteocalcin (ng/mL)	21.059 ± 2.277	24.377 ± 2.678	−4.029	**0.005**	−1.424	[−2.409, −0.395]
Testosterone (ng/mL)	235.423 ± 79.326	133.160 ± 27.062	4.200	**0.004**	1.485	[0.433, 2.493]

Note: Pre = Pre-match measurement; Post = Post-tournament measurement; CI = Confidence Interval; CK = Creatine Kinase; LDH = Lactate Dehydrogenase; IGF-1 = Insulin-like Growth Factor 1; IL-6 = Interleukin-6. Values are presented as mean ± SD. *p* values represent paired-samples *t*-test results. Effect size (d) is calculated using Cohen’s d. Significant results (*p* < 0.05) are shown in bold.

## Data Availability

The data obtained in this study are presented in tables within the article. The raw data are available from the corresponding author upon reasonable request. The data are not publicly available due to ethical and privacy restrictions.
